# The Successes and Challenges of Implementing Telehealth for Diverse Patient Populations Requiring Prenatal Care During COVID-19: Qualitative Study

**DOI:** 10.2196/32791

**Published:** 2022-03-30

**Authors:** Ruth Farrell, Christina Collart, Caitlin Craighead, Madelyn Pierce, Edward Chien, Richard Frankel, Brownsyne Tucker Edmonds, Uma Perni, Marissa Coleridge, Angela C Ranzini, Susannah Rose

**Affiliations:** 1 Obstetrics and Gynecology and Women's Health Institute Cleveland Clinic Cleveland, OH United States; 2 Genomic Medicine Institute Cleveland Clinic Cleveland, OH United States; 3 Center for Bioethics Cleveland Clinic Cleveland, OH United States; 4 Research Innovation and Education Cleveland Clinic Lerner College of Medicine Cleveland, OH United States; 5 Department of Obstetrics and Gynecology Indiana University Indianapolis, IN United States; 6 Department of Obstetrics and Gynecology MetroHealth Medical Center Cleveland, OH United States; 7 Center for Patience Experience Cleveland Clinic Cleveland, OH United States

**Keywords:** prenatal health care delivery, telehealth, COVID-19, patient experience, challenge, telemedicine, pregnancy, women, diversity, prenatal, obstetric, reception, experience, development

## Abstract

**Background:**

Although telehealth appears to have been accepted among some obstetric populations before the COVID-19 pandemic, patients’ receptivity and experience with the rapid conversion of this mode of health care delivery are unknown.

**Objective:**

In this study, we examine patients' prenatal care needs, preferences, and experiences during the COVID-19 pandemic, with the aim of supporting the development of successful models to serve the needs of pregnant patients, obstetric providers, and health care systems during this time.

**Methods:**

This study involved qualitative methods to explore pregnant patients’ experiences with prenatal health care delivery at the onset of the COVID-19 pandemic. We conducted in-depth interviews with pregnant patients in the first and second trimester of pregnancy who received prenatal care in Cleveland, Ohio, from May to July 2020. An interview guide was used to probe experiences with health care delivery as it rapidly evolved at the onset of the pandemic.

**Results:**

Although advantages of telehealth were noted, there were several concerns noted with the broad implementation of telehealth for prenatal care during the pandemic. This included concerns about monitoring the pregnancy at home; the need for additional reassurance for the pregnancy, given the uncertainties presented by the pandemic; and the ability to have effective patient-provider discussions via a telehealth visit. The need to tailor telehealth to prenatal health care delivery was noted.

**Conclusions:**

Although previous studies have demonstrated that telehealth is a flexible and convenient alternative for some prenatal appointments, our study suggests that there may be specific needs and concerns among the diverse patient groups using this modality during the pandemic. More research is needed to understand patients' experiences with telehealth during the pandemic and develop approaches that are responsive to the needs and preferences of patients.

## Introduction

Telehealth is a rapidly evolving approach to the delivery of health care, and significant strides have been made in its use for the delivery of prenatal care [1-4]. The appeal of telehealth is growing, as it provides not only a convenient and cost-effective way to deliver prenatal care, but also a way to improve patient experience [1,5-7]. Although telehealth was incrementally implemented before the emergence of COVID-19 across different patient populations and specialties of medicine, the pandemic radically altered both the timeline and the trajectory of its use to prevent the spread of the virus among health care providers, patients, and communities.

Preventing the spread of COVID-19 has been a particular priority in delivering prenatal care, because prior infectious disease threats, such as H1N1 and Zika, have had major implications for pregnant patients and infants [8-10]. In addition, as there is limited information about the impact of SARS-CoV-2 on pregnant women and newborns, health care institutions made tremendous efforts to ensure social distancing and infection prevention among this population [11-15]. Consequently, there has been rapid adoption of telehealth across diverse obstetric patient populations and health care systems [3].

As a result of initial efforts to implement telehealth in prenatal care, it was possible to begin to operationalize telehealth strategies that allowed patients to continue accessing health care during the pandemic, while eliminating the exposure risks of presenting to an outpatient center or hospital setting. Yet, this required instituting widespread acceptance of this approach among obstetric patient and provider populations who otherwise might not have embraced this type of visit before. For example, some providers either may not have used or elected to use telehealth prior to the pandemic. In addition, some patients may have had limited access to the equipment, internet services, or other resources needed to use telehealth; such issues have often been observed among patients from minority or low-socioeconomic groups [16,17]. Moreover, some patients may not have had an insurance plan that would cover the cost of a telehealth visit, while others may have elected not to use telehealth services because of a preference for in-person visits.

Consequently, although telehealth appears to have been accepted among some obstetric populations before the pandemic, patients' receptivity and experience with the rapid conversion to telehealth are unknown. Furthermore, it is unknown how patients will respond to telehealth visits for obstetric management amidst all the uncertainties presented by the pandemic. It is critical to understand not only patients' experiences with this mode of prenatal health care delivery during the pandemic but also how those experiences may influence health care delivery in the future. Therefore, we conducted a study to understand patients' prenatal care needs, preferences, and experiences during the COVID-19 pandemic to support the development of successful models to serve the needs of pregnant patients, obstetric providers, and health care systems during this time.

## Methods

This study was developed as a qualitative study to explore emerging concepts and themes as they relate to obstetric health care delivery and patient experience during the pandemic.

### Ethics Approval

All research procedures were reviewed and approved by the (deidentified) institutional review board (number 20-408; IRB) of the outpatient clinics of the (deidentified) health care system. Eligible participants were contacted using an IRB-approved study recruitment letter with instructions to contact the research team if interested in participation and informed consent obtained from them.

### Recruitment

We conducted in-depth telephone interviews with pregnant women to understand their prenatal care needs, preferences, and experiences during the COVID-19 pandemic. Inclusion criteria included women who were 18 years of age or older, were English speaking, were able to provide informed consent for research participation, received obstetric care at 1 of the outpatient clinics of the (deidentified) health care system, had a viable intrauterine pregnancy, and had not experienced pregnancy loss at the time of study participation. Recruitment was structured to seek input from 2 groups of women who represent patients at different significant time points in pregnancy. One group included women in the first trimester of pregnancy to capture prenatal care needs, preferences, and experiences at the onset of the pregnancy and prenatal care delivery. A second group included women in the second trimester, whose prenatal care had been established prior to the declaration of a COVID-19 pandemic in the state of Ohio (March 2020). We conducted interviews from May to July 2020. During this time, telehealth was instituted across the health care system and, while encouraged, not required. After providing informed consent, participants took part in a semistructured telephone interview. The interviews were conducted using an interview guide that contained probes to explore pregnant patients' knowledge and perception of the COVID-19 pandemic and the pandemic's impact on their ability to access different aspects of prenatal care. The interview guide was developed in conjunction with content experts in obstetrics, health care communication, medical decision making, clinical genetics, and maternal-fetal medicine. It was piloted before use; the final version was modified based on initial participant feedback. Interviews were audio-recorded and transcribed for analysis.

### Statistical Analysis

Analysis was approached as an iterative and progressive process of data immersion, coding, memoing, and theme identification, an inductive process consistent with grounded theory [18,19]. We identified content domains and categories in transcripts to create a coding tree used to organize the data. A companion codebook was created to serve as a reference for the analysis. The coding and analysis processes were led by 2 members of the study team (authors RF and MP) using NVivo version 12 (QSR International). The research team held weekly meetings to review data coding and memoing and identify themes. Themes identified were contextualized with information about the trimester of pregnancy, gravity/parity, and previous pregnancies.

## Results

### Patient Characteristics

We contacted 115 (first trimester) and 139 (second trimester) patients scheduled at various outpatient clinics within this health care system. Of those, we recruited a total of 40 (15.7%) pregnant women: 20 (50%) in their first trimester and 20 (50%) in their second trimester. The mean patient age was 32.25 (SD 4.54) years, this was the first pregnancy for 15 (37.5%) patients, 3 (7.5%) patients had symptoms of COVID-19 during this pregnancy, 5 (12.5%) were tested for COVID-19, and all results were negative (Table 1). Qualitative analysis identified 4 major themes: (1) perceptions of the benefits of telehealth during the pandemic, (2) the need for reassurance that comes from in-person clinical visits with an obstetric provider, (3) added concerns about being responsible for determining the well-being of the pregnancy at home, and (4) the impact of telehealth on patient experience with pregnancy and prenatal care (see Table 2 for additional qualitative data). No major differences in thematic identification were noted among women in the first trimester of pregnancy (study ID denoted by G1) and women in the second trimester of pregnancy (study ID denoted by G2).

**Table 1:** Demographics (N=40).

Demographics of participants		Participants, n (%)
**Age (years)**		
	Non-AMA^a^ (<35)	27 (67.5)
	AMA (≥35)	13 (32.5)
**Race**		
	White	34 (85)
	Black	4 (10.0)
	Asian	2 (5.0)
**Reproductive history**		
	Primigradiva	15 (37.5)
	Multigradiva	25 (62.5)
**Trimester of pregnancy**		
	First trimester	20 (50.0)
	Second trimester	20 (50.0)
**Tested for COVID-19**		
	Yes	5 (12.5)
	No	35 (87.5)
**COVID-19 results (N=5)**		
	Positive	0 (0)
	Negative	5 (100)
**Use of telehealth (prior to interview)**		
	In-patient visit only	32 (80.0)
	Telehealth visit	8 (20.0)

^a^AMA: advanced maternal age.

**Table 2:** Supplemental qualitative data.

Theme	Illustrative Quotes
Theme 1: Perceptions of the benefits of telehealth during the pandemic	“Truthfully, for a lot of the early prenatal visits, you just sat in the waiting room for what felt like 10 hours, you peed in a cup, and you waited in the exam room for 10 hours. Then, it was a quick Doppler belly check, a quick ‘How are you doing?’, [a] blood pressure check, [and] ‘All good. Great. We’ll see you in a month.’ So that’s it. I can do the blood pressure at home. I could do the Doppler, theoretically. I could do weight at home.” [G1, patient 13] “Well, I think it is very cool that they’re doing virtual visits. One thing that I thought was really neat is that, at my doctor’s appointment, they gave me a Doppler so I can listen to the baby’s heartbeat at home . . . I was very pleasantly surprised. I felt like, you know, unless there’s something major going on, that a virtual visit every now and then was totally appropriate.” [G1, patient 12] “Virtual visits are a big deal . . . I was comfortable doing one. Of course, if there was something I was concerned about it, I could come in. I think it is just less enjoyable to come in because, you know, in the back of your head, you are worried about the possibility of being exposed to someone, especially if you are in a medical building.” [G2, patient 4] “I feel great about it. They sent me home with a blood pressure cuff and I want to say . . . a Doppler. I think that, with the baby and stuff, it really . . . you minimize your exposure, and you don’t have to drive back and forth and sit in a waiting room. So, I feel like as long as you’re healthy and everything’s good, then these are great options.” [G2, patient 10]
Theme 2: Reassurance that comes from in-person clinical visits with an obstetric provider	“She [her provider] said she’d send us home with Dopplers to listen to the baby’s heart at home, which is like, ‘Woah. I’m supposed to sit there and try to find it?’ I can’t even think about that. So, it’s definitely weird and different and not what I expected at all. It makes me nervous that the doctor won’t be right there to do it for me, like someone who went to school for this and is trained in this.” [G1, patient 3] “I don’t necessarily feel a virtual visit with my provider offers the same thing that an in-person appointment would, for example, with measuring your fundal height or making sure the heart rate is good. Unless you have the tools at home or the knowledge, you wouldn’t have the ability to make sure you’re on track compared to your provider doing that in person.” [G1, patient 10] “I would like more in person visits versus the telehealth, just for my own peace of mind that the pregnancy is progressing as it should.” [G2, patient 12] “I just feel like it would feel safer for me to actually see a doctor and actually have them look at me, instead of having the virtual visit. I was never worried or concerned about going to the doctor to get checked.” [G1, patient 8] “Being the first child, I just think it’s nice going into the doctor especially early on when, even though you might have symptoms like you’re tired or nauseous, I don’t have much of a stomach yet, I don’t feel the baby kicking. So, it’s nice to go in and have that reassurance that [the] baby is still growing properly and everything. So, I would prefer going in.” [G1, patient 11]
Theme 3: Added concerns about the responsibility of determining the well-being of the pregnancy at home	“The only thing I would be worried about is if something was wrong. Like, they need to make sure they hear a heartbeat or something like that having more fetal movement when I was further along.” [G1, patient 4] “I went home, and I tried to use it, and it was a couple days later and I couldn’t get it to work, and I was worried it was because there was nothing there to listen to.” [G1, patient 13]
Theme 4: The impact of telehealth on patient experience with pregnancy and prenatal care	“But I think it’s also important to be able to do the in-person visits, and there’s some things I would imagine could get missed just by not having that in-person contact, so I see why that’s important as well.” [G1, patient 14] “Right now, my regular physician has been going teledoc, so we’ve had to do a couple of those. The worst part about it is maybe the connection when you’re standing at a laptop and there might be, like, a delay. So, that is just something you gotta take, I guess, if you’re doing virtual. I wouldn’t not be interested in it.” [G2, patient 9] “I don’t necessarily feel a virtual visit with my provider offers the same thing that an in-person appointment would.” [G1, patient 10]

### Perceptions of the Benefits of Telehealth Visits During the Pandemic

For some participants, their first exposure and opportunity to use telehealth were because of the changes made in response to the pandemic. Some discussed the convenience of this approach. As described by 1 participant who had not elected for telehealth visits before the pandemic,

I realized that the only things that were different with a virtual visit would be checking the heart rate and checking the blood pressure. Once I realized I wasn't really missing out on much and that I can check the heart rate at home by myself, made me feel better about them [telehealth visits]. I wasn't missing out on anything.

G2, patient 2

This finding was noted among participants in the first trimester who were beginning their prenatal care and participants in the second trimester who had already had a series of in-person visits with their provider prior to the pandemic.

Yet, for many, the benefit of telehealth was the ability to reduce the risk of exposure to COVID-19. A recurrent subtheme was concern about the “the possibility of being exposed to someone” [G2, patient 2] while presenting for an in-person prenatal care visit. For some, “virtual visits” (the term used for telehealth in this health care system) felt “safe” and “safer” than going into the office during the pandemic to avoid unnecessary potential exposures during the visit for themselves and their family. Although many participants noted the steps health care clinics and providers had taken to prevent COVID-19 exposure, there was a concern about other patients who presented for care.

I think that the home virtual visits are a really great option so I don't have to sit in the waiting room. Even though everyone has a cloth mask on, it's just a nice comfort level to avoid unnecessary exposures to who knows what. [. . . ] I really looked forward to my prenatal visits in my other pregnancies. But, sitting in the waiting room with everybody and masks on, and seeing the front door be so busy with people, it just made me more aware of all the people that are touching surfaces and adding to the potential exposure. Because, you can't really, really trust all people.

G1, patient 13

For these participants, telehealth visits added a degree of safety and reassurance.

### The Need for Reassurance That Comes From In-Person Clinical Visits With an Obstetric Provider

Although some participants spoke of the benefits of telehealth during the pandemic, others discussed reservations with this approach. Several participants discussed how they weighed their needs and preferences for reassurance with an in-person visit against the risk of possible COVID-19 exposure by presenting to an office or health care center.

It would feel safer for me to actually see a doctor and actually have them look at me instead of having the virtual visit.

G1, patient 8

Participants were aware of the risks of COVID-19 for themselves and their families. Yet, many also felt increased uncertainty from the pandemic and stated a preference for in-person visits to alleviate that uncertainty. As described by this participant who initiated prenatal care during the initial phases of the pandemic,

When I first found out I was pregnant, it was scary and stressful. When I found out, that's when the whole pandemic started. So, it was very, very stressful at first. The only thing that we were thinking of was I have a way to protect myself [. . .]. I just feel like it would feel safer for me to actually see a doctor and actually have them look at me instead of having the virtual visit. I was never worried or concerned about going to the doctor to get checked.

G1, patient 8

The preference for an in-person visit was noted among women in the first and second trimester of pregnancy. Some participants in the first trimester of pregnancy preferred in-person visits to reassure them of the status of the pregnancy, particularly before there were visible or tangible changes of pregnancy. This reassurance is something that they did not feel could come from a telehealth visit. As described by this participant in the first trimester,

I wanted to make sure I was still pregnant even though I took like ten tests and they all said, “Yes.” I just definitely wanted to make sure I still am . . . I want to make sure everything's gonna be okay.

G1, patient 3

This was also observed among women in the first trimester who had never been pregnant.

My doctor said that, maybe down the line, we could do some virtual visits. I thought, oh, that's interesting. I've never done those. I would save some time and energy on those. But at the same time, I still feel like, with my first pregnancy, I should still get to the office and have [one-on-one], face-to-face.

G1, patient 7

This was a theme noted across both trimesters. For example, participants in the second trimester also sought reassurance until they could experience tangible evidence of the pregnancy.

I still wanted to go into the doctor at least until I could feel the baby moving because I don't feel many symptoms when I'm pregnant. So, I just wanted confirmation that there was still a baby in there.

G2, patient 21

### Added Concerns About Responsibility for Determining the Well-being of the Pregnancy

Participants also spoke of their concerns about managing their prenatal care with home monitoring and during their telehealth visit. These concerns augmented existing concerns about their health and the well-being of the pregnancy during the pandemic. One main subtheme was that although participants were provided with the instructions and tools to check the presence and status of the fetal heart tones, they noted some concern over whether they had the skills and competence to use the equipment. This concern stemmed from an underlying concern that “something would be missed” or “overlooked” if a trained health care provider did not perform the evaluation in person.

So, they gave me a blood pressure monitor and a Doppler. So, I guess, I am going to be in charge of doing those things, which is making me anxious.

G1, patient 2

Participants worried both about missing a potential health problem and about misinterpreting a result so that they would suspect a problem that did not really exist. As described by this patient,

So, my OB [sic] also does the virtual visits, and they said, “We'll give you a blood pressure cuff. We'll give you a Doppler. You can check your own baby's heart rate.” And to me, I mean I know they're taking precautions, everybody . . . all the doctors are trying to see less patients and all that. But I feel like, being pregnant, I feel like that should have to be something an OB [sic] should have to physically evaluate their patient for, not me doing it myself. [. . .] I just feel like it's an important thing that the OB should be the one to physically assess the baby and me. Check my blood pressure for me because I'm not . . . I'm not trained in that. I feel like that's their job and they're the ones that are the trained professionals and they would know how to do that better than I would.

G2, patient 17

Several participants also had a profound fear of discovering an obstetric complication or pregnancy loss while using the equipment provided for at-home monitoring. As described by this participant who was provided a Doppler to hear the fetal heart tones,

The Doppler . . . I tried using it a couple times, [and] it's still early on in the pregnancy, so it is harder to pick up on. Sometimes I'm like, “Oh gosh. Why don't I see it? Is something wrong?”

G1, patient 11

This was also a prominent concern among participants who had experienced a pregnancy loss or complication in an earlier pregnancy. As described by this participant,

I know my next appointment will be virtual and I'll need to do my own Doppler, and that's definitely different. So, I'm not sure how I'll feel about it, especially with the scare we had [referring to a threatened miscarriage]. I'm not sure if I can't find it [heartbeat], or with me being early on, if I can't find something. It would be a huge worry on me.

G1, patient 6

### The Impact of Telehealth on Patient Experience

The meaning and significance of the experience of pregnancy were an important topic for participants. Participants sought meaningful and personal experiences with the pregnancy, something many felt that in-person access to a health care provider could offer.

If I don't go [into the office], I don't get to see the progress of my baby, and I want to be able to see that still despite the pandemic.

G1, patient 20

There was also a sense of confronting and managing a sense of loss in the expected experience and the reality of prenatal care as it was unfolding during the pandemic.

I never imaged my first pregnancy would be like this and that I'd be talking to my doctor about a lot of things over the phone or with a mask on in our visits.

G1, patient 3

Participants spoke of the desire to have an experience where they could align their care with their personal needs and preferences during pregnancy. Some participants noted that communication during a telehealth visit was different than their expectations for their prenatal care or than their experiences during another kind of telehealth visit. For participants, there seemed to be greater familiarity or effectiveness during an in-person interaction with the health care provider.

I prefer in-person visits because sometimes, I think of more questions to ask or I have certain concerns. So, I feel like I get better answers and support in-person.

G1, patient 5

Interpretation of nonverbal communication, gestures, body posture, and facial expressions was also a concern for telehealth visits. As this participant explained,

I do wonder if you'll be able to catch everything that you would have [at] an in-person appointment. I know you go through your basics. You go through your basic numbers like the fetal heart rate, blood pressure, and such. [. . .] In person, with facial expressions and such, you can pick up on some subtleties. So, I do wonder if something might get lost.

G2, patient 23

For some patients, the personal nature of pregnancy, with discussions of reproduction, children, and family, presented a sharp contrast to other types of medical visits. This participant described the uniqueness of obstetric visits and conversations compared to other health care encounters:

But it is a . . . it's a very personal thing, and I think that you kind of need to be with your doctor physically. I think it's just a different kind of care. It's a different kind of appointment, and so, I think it's a mistake to treat OB/GYN care just like any other doctor's appointment, like, ‘cause, I just think it's different in so many ways.

G2, patient 18

## Discussion

### Principal Findings

Our study provides important insight into the nature and scope of health care delivery challenges caused by the COVID-19 pandemic, specifically those associated with the implementation of telehealth as a formative strategy to control infection spread among health care providers, patients, and communities. Although previous studies demonstrated that telehealth is an acceptable and available way to obtain prenatal care for some pregnant patients [2,6,20], the majority of these studies were conducted prior to the pandemic and with patient populations who may have been more accepting of or able to use this approach. As a result, there is little data on the impact of broad and rapid telehealth use across diverse patient and health care provider populations in response to a pandemic.

Our study sheds light on patients' experiences and attitudes about integrating telehealth in prenatal health care delivery. Many participants in this study supported the use of telehealth in their prenatal care, citing reasons, including a sense of greater convenience, less time commitment, and less frustration from logistic steps of navigating the clinical setting compared to in-person visits; similar reasons had been noted in previous studies on the use of telehealth. In addition, they valued telehealth as a way to avoid possible exposure to COVID-19 and the potential negative consequences of infection for themselves, the pregnancy, and their family. Such factors may help patients who may be unsure of or inexperienced with using the internet for health care to try telehealth [21-25].

However, there were several concerns about telehealth broadly and its rapid integration during the pandemic, concerns that have not been reported in earlier studies of the use of telehealth in prenatal health care delivery. A leading finding was that many participants sought additional reassurance about their health care during the pandemic, particularly the implications of COVID-19 for the pregnancy and the best approaches to managing key aspects of their pregnancy amidst the pandemic (eg, accessing testing during pregnancy, management of labor and delivery). Several participants felt that they could only achieve the level of reassurance they needed from an in-person visit. This was due, in part, to a concern that health care communication may not be as effective during an internet-based interaction compared to an in-person visit. Participants described how they viewed the use of telehealth for obstetric care compared to its use for general health care concerns. Reasons included the need for additional care and sensitivity needed for personal discussions pertaining to reproductive health, the pregnancy, parenthood, and family. For these participants, different and unfamiliar approaches to patient-provider communication also led to questions about health care quality and patient experience during their pregnancy as it was uniquely affected by the ongoing pandemic. Among this group, there was a call for health care systems to adapt telehealth approaches that would address the nuances of pregnant patients and prenatal care delivery.

Other important concerns were described. As part of telehealth implementation, participants were often provided with home monitoring equipment, such as a fetal Doppler to assess the fetal heartbeat at home. Most felt satisfied with the education provided by their health care provider about how to use this equipment. Yet, several were concerned about using this equipment at home [26,27], specifically whether they had the ability to correctly use the Doppler to hear the heart tones. This is a unique finding that calls for further investigation as other studies show patients' acceptance of using home monitoring tools [28]. Yet, a recent study demonstrated that 68% of participants were comfortable using a fetal Doppler for fetal heart tones [26], raising a question of the perspectives of those participants who did not. Further research is needed to understand how diverse patient populations may be able to implement home monitoring plans as part of telehealth, an issue that may be affected by health literacy and other key patient demographic factors.

Another concern pertained to a fear that they would be the ones to directly identify miscarriage or fetal loss by detecting the lack of a heartbeat using the fetal Doppler. Prior studies demonstrate the fears and experiences of women regarding the experience of miscarriage, particularly among those who experienced loss in a prior pregnancy [29-33]. These are significant considerations that must be addressed in telehealth implementation, both for patients experiencing pregnancy during the pandemic and for those who may use telehealth under different circumstances.

These findings raise questions about how patient experience and health care communication, key metrics to health care quality and safety, may be altered by telehealth, particularly when broadly implemented during a pandemic or other similar public health crises. Obstetric patients may have unique needs with the implementation of telehealth during the pandemic, given the myriad of uncertainties that COVID-19 has introduced to health care deliveries and communities. A model by Peahl et al [6] suggested that a 4-1-4 method may be possible for prenatal care, describing a process that combines in-person with telehealth visits that may balance the preferences and needs of patients, while also maintaining health and safety during an infectious public health threat. Thus, there is a need to develop approaches to telehealth for prenatal care delivery that align with the preferences and needs of diverse pregnant patient populations.

### Limitations

Although our study sheds light on the emerging challenges with telehealth implementation, there were some limitations. The study used qualitative methods among a sample of patients at a single health care system. Our sample was limited in size and in racial and ethnic representation among diverse populations. In addition, study participants were from a health care system that could readily deploy telehealth, which may reflect participants' interest and exposure to this approach. Therefore, further research is needed to examine study findings among diverse patient populations and as the course of the pandemic continues.

### Conclusion

In conclusion, although previous studies have demonstrated that telehealth is a flexible and convenient alternative for some prenatal appointments, our study suggests that there may be specific needs and concerns among the diverse patient groups using this modality during the pandemic. More research is needed to understand patients' experiences with telehealth during the pandemic and to develop approaches that are responsive to both the needs and preferences of patients and the challenges presented by public health emergencies that call for significant changes in health care delivery. This includes understanding the experiences of not only pregnant patients but also patient populations who may also have experienced the rapid conversion to telehealth in response to the pandemic.

## Abbreviations

IRB: institutional review board
